# MRI measurement of cerebral perfusion in severe congenital heart disease: just the first step

**DOI:** 10.1038/s41390-024-03300-6

**Published:** 2024-06-07

**Authors:** Kevin B. Kilgallon, Ira M. Cheifetz

**Affiliations:** Department of Pediatrics, Rainbow Babies and Children’s Hospital, Case Western Reserve University School of Medicine, Cleveland, OH USA

Two questions on the mind of many parents of children with complex congenital heart disease (CHD) are: Will my child survive? And, how will their life be affected? As diagnostic procedures, along with medical and surgical treatments, for these infants have improved over the recent decades, more and more of these children are surviving into adolescence and adulthood. Despite these advances in care, children with complex CHD are at higher risk of neurodevelopmental disability (NDD) and decreased quality of life (QOL) compared to their peers without CHD.^1,2^

As many as 50% of children with complex congenital heart disease will exhibit NDD, making these issues more common than all cardiovascular-related morbidity combined. NDD includes cognitive impairment, gross and fine motor problems, social communication difficulties, behavioral and psychological problems, including ADHD, and executive function impairments, with these conditions often co-occurring.^3–6^ Werninger and colleagues have reported a correlation between MRI findings and NDD in children, adolescents, and adults with complex CHD.^6^ While these disabilities are often mild, they can affect educational achievement, vocational outcome, daily function, and overall QOL.^2,7^ The mechanisms of abnormal brain development are wide ranging and include prenatal and postnatal alterations in blood flow (cerebral and systemic), compromised oxygen delivery, and likely inflammatory effects throughout the body. Potential insults can be perioperative, including adverse effects of cardiopulmonary bypass and circulatory arrest, and postoperative, including prolonged hospital stays, pharmacologic sedation and neuromuscular blockade, feeding difficulties with associated alterations in nutritional status, and family psychosocial stresses.^1,2,8^ Studies in multiple high risk pediatric populations demonstrate that early interventions can improve morbidity and neurodevelopmental outcomes, but a critical first step is recognizing which children are at increased risk and why.^9–11^

In this issue, De Silvestro and colleagues sought to identify changes in cerebral blood flow and oxygen delivery in infants with different types of severe CHD and compare them to healthy controls.^12^ They used pseudocontinuous arterial spin labeling MRI (pCASL) to quantify cerebral blood flow to cortical and deep gray matter. Severe CHD was defined as requiring surgery within the first 6 weeks of life. Cerebral perfusion was compared between CHD and healthy controls, and the effects of systemic to pulmonary shunt, aortic arch obstruction, and arterial oxygen saturations were examined along with the possible mechanistic effects on cerebral blood flow. These investigators discovered that in children with severe CHD, the presence of a systemic to pulmonary shunt decreased perfusion to cortical gray matter, and perfusion decreased proportionally as shunt size increased. Arch obstruction and arterial oxygen saturation did not alter cerebral perfusion. Interestingly, cerebral oxygen delivery was unaffected by either a systemic to pulmonary shunt or an arch obstruction. Notably, children with severe CHD had increased cerebral perfusion as compared to healthy controls when adjusting for the presence of a systemic to pulmonary shunt, arch obstruction, and arterial saturation, with preserved cerebral oxygen delivery.^12^

This study is important in a number of ways. It marks a vital step toward understanding one of the key mechanisms of neurodevelopmental changes in children with complex CHD, namely alterations in cerebral blood flow and oxygen delivery both pre- and post-operatively. These effects begin even in the fetal stage as pathologies such as left-sided obstructive lesions and transposition of the great arteries affect the flow of blood to the brain and the saturation of the blood in the upper and lower body during fetal circulation.^13,14^ After birth, a systemic to pulmonary shunt can cause steal of blood from the cerebral circulation and arterial oxygen saturation can affect cerebral oxygen delivery.^14^ The authors showed that systemic to pulmonary shunts do alter cerebral blood flow, and that larger shunts magnify this effect, but aortic arch obstruction does not. Furthermore, children with severe CHD seem to have increased cerebral perfusion compared to their healthy peers when correcting for presence of a systemic to pulmonary shunt, arch obstruction, and arterial oxygen saturation. This demonstrates additional strong evidence for the brain’s ability to alter cerebral blood flow to maintain oxygen delivery.^12^

The findings from this study are critical to our understanding of the effects of CHD on brain blood flow and subsequent neural development. However, this is just a first step as there are several important questions to answer now that we are armed with this knowledge. The brain’s ability to maintain cerebral oxygen delivery is remarkable. Indeed, this is a key reason that children born with severe CHD and undergo neonatal cardiac interventions are able to survive, let alone generally thrive. Why then, are there still such high rates of NDD, and why are certain children at higher risk than others? We can investigate using pCASL to compare larger groups of patients and analyze them by specific lesions (e.g., transposition of the great arteries, hypoplastic left heart syndrome, tetralogy of Fallot, etc.). Taking this a step further, MRI can be used to measure not only blood flow and oxygen delivery, but oxygen extraction as well, thus, allowing us to more thoroughly investigate the alterations in cerebral energy metabolism that may lead to changes in brain development.^15^ Evaluating cerebral blood flow in white matter in addition to gray matter is important as well, as white matter injuries are known to be an important component of brain alterations in CHD.^16,17^

Once more granular data on cerebral oxygen delivery and metabolism are obtained, we should then be able to follow these children longitudinally to monitor neurodevelopment and link specific changes in cerebral blood flow and energy metabolism to neurodevelopmental outcomes. Survival with good functional and developmental outcome is, of course, paramount when caring for these children and counseling families If we were able to directly tie specific lesions to changes in cerebral blood flow and subsequent long term outcomes, we could better focus our efforts to improve these outcomes with early goal-directed therapies, family support, and even changes in surgical and medical treatments (e.g., comparing Sano shunt to Blalock-Taussig-Thomas shunt to hybrid procedure).

Looking in the other direction, another question is how does bedside monitoring correlate with cerebral blood flow and oxygen delivery? In the pediatric cardiac critical care setting, patients are routinely monitored for systemic oxygen saturation, blood pressure, and cerebral near infrared spectroscopy (NIRS). If we could use MRI to quantify cerebral blood flow and relate it to these commonly monitored bedside parameters, it could provide us an opportunity for immediate intervention to improve cerebral oxygen delivery. We may then feel more confident knowing that changes in our vasoactive medications and fluid management to improve a trend in cerebral NIRS can not only reverse developing metabolic acidosis but can also improve a child’s ability to grow and develop as normally as possible. Such beneficial outcomes would follow these children into adulthood.

This type of work could eventually provide us a powerful axis of information, relating changes in bedside monitoring to MRI-quantified cerebral perfusion and oxygen delivery and then subsequently to long term positive neurodevelopmental outcomes. Such an approach could reflect a paradigm shift in caring for children with CHD and provide important data to allow incremental and consistent improvement in their overall care.

The fields of pediatric cardiology, critical care, and cardiac surgery are both difficult and rewarding, as we are often providing care that saves the lives of children with severe CHD while at the same time setting some on a path of lifelong hardship in the form of NDD and repeat hospital stays. This study by De Silvestri and colleagues provides a blueprint to potentially develop new ways to improve the lives of our patients. The future of our field is an exhilarating one; although we have come so far, we still have so much farther to go. Chinese philosopher Lao Tzu stated, “a journey of a thousand miles begins with a single step;” studies such as this could be that step.
